# Sequence analysis of fusion protein gene of Newcastle disease virus isolated from outbreaks in Egypt during 2006

**DOI:** 10.1186/1743-422X-8-237

**Published:** 2011-05-18

**Authors:** Mahmoud HA Mohamed, Sachin Kumar, Anandan Paldurai, Siba K Samal

**Affiliations:** 1 Faculty of Veterinary Medicine, Zagazig University, Zagazig, Egypt; 2 Virginia-Maryland Regional College of Veterinary Medicine, University of Maryland, College Park, MD 20742, USA

**Keywords:** Newcastle, disease virus, African strain, Chickens, Sequence

## Abstract

**Background:**

Newcastle disease virus represents APMV-1 and is the most characterized among all APMV types. The F protein cleavage site sequence is a well-characterized determinant of NDV pathogenicity in chickens. In this study, the sequences of fusion protein (F) gene of three Newcastle disease virus (NDV) strains isolated from outbreak in chickens in the Al-Sharkia province of Egypt in 2006 were determined.

**Findings:**

The viral genomic RNAs were extracted from the infective allantoic fluid and F gene is amplified using primer sets designed from the available sequences of NDV strains from GenBank. The pathogenicity of NDV strains was determined by three internationally recognized tests mean death time, intracerebral pathogenicity index, and intravenous pathogenicity index. The phylogenetic analysis showed that the Egypt isolates are closely related with the genotype II of class II NDV strains.

**Conclusions:**

The sequences of the F genes of the 2006 Egypt isolates are closely related to that of the 2005 Egypt isolate from the same province suggesting that these strains are probably circulating in the vaccinated bird population in Egypt until development of an outbreak.

## Findings

Newcastle disease (ND) is a highly contagious and fatal disease of chickens. In many developing countries ND is endemic and the disease has the greatest impact on villages where the livelihood of people depends on poultry farming. The causative agent Newcastle disease virus (NDV), is a member of the genus *Avulavirus *in the family *Paramyxoviridae*. NDV isolates display a spectrum of virulence in chickens ranging from inapparent to fatal infection. Based on their pathogenicity in chickens, NDV isolates are categorized into three main pathotypes: lentogenic (low virulence), mesogenic (intermediate virulence) and velogenic (high virulence). It may appear that continents having warm climates are reservoirs of virulent NDV strains [1]. NDV is a well-studied paramyxovirus and complete genome sequences of many North American NDV strains are available; however, very little is known about the genome sequences of NDV strains isolated from different parts of Africa with in isolated cases [2-4].

ND outbreaks occur frequently in Egypt and the source of the virulent NDV in these outbreaks are not known. We have reported the complete genome sequence of a NDV strain isolated from an outbreak at a poultry farm in Al-Sharkia province, Egypt in 2005 (chicken/Egypt/1/2005) [5]. In this study, we have determined the fusion (F) gene sequences of three NDV strains isolated from outbreaks on poultry farms in Al-Sharkia province, Egypt in 2006. The infected birds showed severe neurological and/or respiratory symptoms. The viruses were confirmed as NDV by hemagglutination inhibition assay using a known NDV antiserum. The pathogenicity of NDV strains was determined by three internationally recognized tests mean death time (MDT), intracerebral pathogenicity index (ICPI), and intravenous pathogenicity index (IVPI) [6]. The NDV strains were designated as NDV/chicken/Egypt/2/2006, NDV/chicken/Egypt/3/2006 and NDV/chicken/Egypt/4/2006. NDV strain LaSota was used as a positive control for all pathogenicity studies. The results of the pathogenicity tests showed that all three NDV strains were velogenic (Table 1).

**Table 1:** Pathogenicity index tests of Egyptian strains of NDV.

Strains	IVPI	ICPI	MDT
NDV/chicken/Egypt/2/2006	2.1 ± 0.24	1.6 ± 0.15	60 ± 1.5

NDV/chicken/Egypt/3/2006	2.2 ± 0.29	1.8 ± 0.19	50 ± 1.8

NDV/chicken/Egypt/4/2006	2.25 ± 0.23	1.7 ± 0.18	55 ± 2.3

NDV/LaSota	ND	0.0	102 ± 2.5

Intravenous pathogenicity index (IVPI) was done in 6-week-old chickens, intracerebral pathogenicity index (ICPI) test in 1-day-old chicks inoculated intracerebrally and mean death time (MDT) in 9-day-old eggs inoculated through allantoic cavity following standard protocol.

The F gene was chosen for sequencing because this gene is a major determinant of virulence and NDV isolates are grouped into genotypes based on the sequences of this gene [7-9]. To determine the F gene sequence of all the Egyptian strains, the viruses were grown in the allantoic cavities of 9-day-old embryonated specific pathogen free (SPF) chicken eggs. Plaque purification of the viruses was not performed to avoid the genetic selection, which may not represent the actual viral genome. The viral genomic RNAs were extracted from the infective allantoic fluid using RNeasy mini kit (QIAGEN). Primer sets were designed from the available sequences of NDV strains Beaudette C (accession numbers X04719) and Texas GB (accession numbers GU978777). Three primers namely F-1for 5' ACGGGTAGAAGATTCTG 3', F-655for 5'GTTGACTAAGTTAGGTG 3'and F-1780rev 5' CTCTCCGAATTGACAGAC 3' (number corresponds to NDV F gene nucleotide sequence) were used to sequence the entire F gene. Reverse transcription and PCR were done using virus specific primers by Superscript-II reverse transcriptase and high fidelity Platinum *Pfx *polymerase (both from Invitrogen), respectively. The PCR-amplified products were directly sequenced using BigDye terminator v 3.1 matrix standard kit and 3130*xl *genetic analyzer data collection software v3.0 (Applied Biosystems Inc). The entire F gene was sequenced at least three times from independent RNA preparations to ensure a consensus sequence. All experiments were carried out in an enhanced BSL3 containment facility certified by the USDA to work with highly virulent NDV strains with the investigators wearing appropriate protective equipment and compliant with all protocols approved by the Institutional Animal Care and Use Committee (IACUC) of the University of Maryland and under Animal Welfare Association (AWA) regulations.

**Figure 1:**
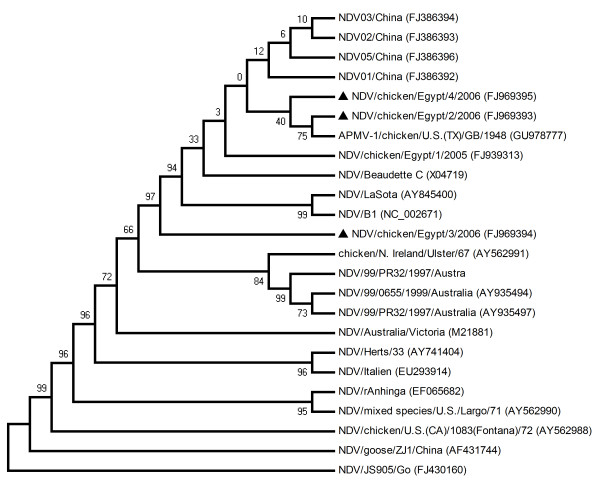
Phylogenetic tree of Egyptian strains with other NDV strains was constructed using maximum parsimony method with bootstrap values calculated for 1000 replicates (Egyptian strains were marked with solid triangle)

The F gene of all three Egyptian strains of NDV is 1792 nt in length and encodes a predicted F protein of 553 amino acids (GenBank under accession numbers FJ969393, FJ969394 and FJ969395). Sequence analysis of the F protein of Egyptian strains showed 94% to 99% amino acid identity with that of strain BC (Table 2). Phylogenetic analysis showed that strains chicken/Egypt/2/2006 and chicken/Egypt/4/2006 are closely related with the virulent NDV strain Texas GB, while strain chicken/Egypt/3/2006 stands out from other NDV strains in genotype II of class II viruses (Figure 1). The F protein cleavage site sequence is a well-characterized determinant of NDV pathogenicity in chickens [8-10]. Virulent NDV strains typically contain a polybasic cleavage site (R-X-K/R-R↓F), which is recognized by intracellular proteases present in most cell types. The cleavage site of all Egyptian strains contained four basic amino acids at positions 112-116 (^112^R-R-Q-K-R↓F-I^118^), corresponding to those of virulent NDV strains similar to the one that was isolated in 2005 from the same province [5]. In addition, the presence of the phenylalanine (F) residue at position 117 has been described as being a possible contributor to the neurological effects [11,12]. Interestingly, the F cleavage site of NDV strains isolated from Egypt was identical to those of NDV strains isolated from chickens and guinea fowl in the Mopti and Sikasso regions of Mali in 2008 and many other NDV strains from China [5,13,14].

**Table 2:** Percent amino acid sequence identity of the Egyptian strains with other strains of NDV.

NDV strains	Egypt/2/2006	Egypt/3/2006	Egypt/4/2006	Egypt/1/2005	LaSota	BC	Fontana
Egypt/2/2006		94.4	98.9	98.4	97.5	99.1	90.4
Egypt/3/2006			94.0	93.3	93.1	93.9	92.6
Egypt/4/2006				97.6	98.2	98.0	90.1
Egypt/1/2005					97.5	99.1	90.8
LaSota						97.8	90.4
BC							91.3
Fontana							

DNA pair-wise alignment was done using MegAlign (clustalW) in a Lasergene 8 software package.

Our results indicate that high levels of nucleotide and amino acid sequence identity exist between the African and North American NDV strains. It is possible that NDV strains isolated from Egypt may represent the strains circulating in the poultry population in that region. These results also suggest that more than one genotype of NDV is circulating in the African subcontinents. However, determination of the complete genome sequences of Egyptian strains isolated in different geographic regions of Africa is necessary to understand the genetic relatedness among NDV strains circulating in different parts of the world.

The close genetic relatedness of the NDV strains isolated in 2005 and 2006 in the same province in Egypt suggest that these strains are endemic in the bird population in this province. Furthermore, these results suggest that NDV vaccine used in this province may not be very effective in stopping viral shedding, which will allow unnoticed circulation of the virulent virus in the vaccinated bird population until development of an outbreak. Therefore, it may be necessary to evaluate the effectiveness of the current vaccine used in the Al-Sharkia province against circulating NDV strains.

## Competing interests

The authors declare that they have no competing interests.

## Authors' contributions

Conceived and designed the experiments: MHM and SKS. Performed the experiments: MHM, SK and AP. Analyzed the data: MHM, SK and AP. Contributed reagents/materials/analysis tools: MHM and SKS. Wrote the paper: MHM, SK and SKS. All the authors have read and approved the final manuscript.
